# Intra- and inter-observer analysis in the morphological assessment of early-stage embryos

**DOI:** 10.1186/1477-7827-7-105

**Published:** 2009-09-29

**Authors:** Goedele Paternot, Johanna Devroe, Sophie Debrock, Thomas M D'Hooghe, Carl Spiessens

**Affiliations:** 1 Leuven University Fertility Centre, UZ Leuven, Campus Gasthuisberg, Herestraat 49, 3000 Leuven, Belgium

## Abstract

**Background:**

The aim of this study was to determine the intra- and inter-observer variability in the evaluation of embryo quality. Multilevel images of embryos on day 1, day 2 and day 3, were analysed using different morphological parameters.

**Methods:**

Multilevel images of embryos on day 1, day 2 and day 3, were analysed using a standard scoring system. The kappa coefficient was calculated to measure intra- and inter-observer variability before and after training sessions.

**Results:**

Good to excellent intra-observer agreement was present for most parameters exceptions being scoring the position of pronuclei and the presence of a cytoplasmic halo on day 1, multinucleation on day 2 and the size of fragments on day 3. Inter-observer agreement was only good to excellent for the number of blastomeres on day 2 and day 3 and the orientation of the cleavage axes on day 2. Training sessions had a positive impact on inter-observer agreement.

**Conclusion:**

In conclusion, assessment of morphological characteristics of early stage embryos using multilevel images was marked by a high intra-observer and a moderate inter-observer agreement. Training sessions were useful to increase inter-observer agreement.

## Background

Reproductive outcome in IVF depends on an interplay of different factors including embryo quality [1,2]. Many different classification and scoring systems, based on cleavage and morphological parameters, have been proposed to evaluate embryo quality [2-4]. However, none of these grading systems has been completely validated [5,6]. Reliability is one of the most important conditions for the validation of a scoring system. Currently, the scoring of cleavage stage embryos involves the direct evaluation of the embryo by an embryologist assessing the morphological parameters to determine embryo quality [1].

Some variation in scoring of morphological parameters can be expected among different embryologists (inter-observer variability) and within the same embryologist (intra-observer variability) [7]. However, only limited and controversial data [8,9] are available with respect to intra- and inter-observer variability in the evaluation of morphological parameters assessing embryo quality. No data are available with respect to this variability by means of multilevel images. The use of these multilevel images allows embryologists to assess embryo quality in the same way as an exploration at the inverted microscope, but without a limitation in evaluation time.

Therefore, the aim of this study was to determine the intra- and inter-observer agreement in the assessment of embryo quality on day 1, 2 and 3 by means of multilevel images.

## Methods

### Image collection

A computer system (FertiMorph, IHMedical A/S, Copenhagen, Denmark) was used to record image sequences of 50 embryos on day 1, day 2 and day 3 of their development. This system allowed investigators to record image sequences that consisted of 26 images of the same oocyte or embryo by automatically focussing through the complete embryo (5 μm intervals). In our study, embryos (N = 50) from 6 IVF/ICSI patients with an oocyte retrieval on the same day were included in order to have a random sample of embryos representing the embryos in daily routine. The mean age of the 6 patients was 29.5 (SD+/-3.27) years. All embryos were evaluated for each parameter.

### Embryo scoring system

The multilevel images were presented to the embryologists on a patient-by-patient basis and shown in a chronological order of embryo development. Embryo quality was scored according to the criteria defined in the Standard Operating Procedures of the Leuven University Fertility Centre (LUFC) scoring system. The following parameters were evaluated: day 1: the size (equal or unequal (>25% difference in size)) and position (central or peripheral) of the pronuclei, the presence of a cytoplasmic halo (a light zone over at least 75% of the circumference of the zygote); day 2 and day 3: the number and size (equal, slightly unequal (25-50% difference in size) or unequal (>50% difference in size)) of the blastomeres, the degree of fragmentation (grade 0: no fragmentation, grade 1: <10%, grade 2: 10-25%, grade 3: 25-50%, grade 4:>50% fragmentation), the position (local or dispersed) and the size (small or large) of the fragments. Furthermore, the position of the cleavage axes (perpendicular or not) on day 2 (if a 4 cell embryo was available) and the presence of mono- or multinucleation on day 2 and day 3 were evaluated. Since there was no visible nucleus in the majority of the embryos on day 3, this parameter was excluded.

### Intra-observer variability

Five embryologists (Table 1) were asked to evaluate the embryos at two different time points with a time interval of minimum one month and maximum two months. Each embryologist was blinded with respect to the assessment of the embryo quality in their first evaluation and to the results of the assessments of embryo quality by the other embryologists.

**Table 1:** Participation (yes/no) of individual embryologists (number of years of experience) to the three components of our study (intra-observer variability, inter-observer variability, training sessions)

**Participant** **(N years of experience)**	**Intra-observer** **variability**	**Inter-observer** **variability**	**Training sessions**
Embryologist 1 (6.33)	Yes	Yes	Yes
Embryologist 2 (5)	Yes	Yes	Yes
Embryologist 3 (3.25)	Yes	Yes	Yes
Embryologist 4 (5.5)	Yes	Yes	No
Embryologist 5 (1.5)	Yes	Yes	No
Embryologist 6 (16)	No	Yes	No
Embryologist 7 (3.25)	No	Yes	No

### Inter-observer variability

Inter-observer variability in embryo scoring was assessed among 7 embryologists (Table 1). Each embryologist was blinded to the assessments of the other embryologists.

### Training session: scoring of "experts" versus "trainees"

In a next step, three trainees who had no experience in the scoring of embryos were asked to score the same embryos with the LUFC scoring system. Only the definition of the parameters used in the LUFC scoring system was given to them as foreknowledge. Two weeks later, these three trainees and three embryologists (with respectively 3.25, 5 and 6.33 years of experience) (Table 1) were given three training sessions in one week. During these three training sessions, embryo images were shown to visually demonstrate the definitions of all parameters. In this way, a consensus was reached regarding the way the parameters had to be scored. Based on these definitions, a manual was created with a clear description and some examples of each parameter. During the two weeks following the training sessions, the same six persons (three trainees and three trained embryologists) scored the embryos again, this time they used the manual in order to assess the impact of training and pre-existing experience on the inter-observer variability in the assessment of embryo quality. The aim of these training sessions was to increase the number of parameters with a kappa coefficient value higher than 0.60.

### Statistics

The kappa coefficient was calculated to measure intra-observer (comparison of embryo scoring given at two different time points by the same embryologist) and inter-observer (comparison of embryo scoring by different embryologists) variability. This kappa coefficient was interpreted as an indicator of either excellent (≥ 0.80), good (0.60-0.79), moderate (0.40-0.59), poor (0.20-0.39) and very poor (<0.20) intra- and inter-observer agreement [10]. The number of observations necessary to do kappa statistics is calculated by the equation: 2n^2^; with n the number of categories for each characteristic. In this study, the degree of fragmentation has the highest number of categories (n = 5) indicating the need for at least 2*(5)^2 ^= 50 embryos [11]. The major disadvantage of using this kappa coefficient is that no statistics are available to compare different kappa values.

## Results

### Intra-observer variability

Excellent intra-observer agreement was observed for the number of blastomeres on day 2 and day 3 and for the orientation of the cleavage axes on day 2 (Table 2). Good intra- observer agreement was observed for the following parameters: position of the pronuclei, the degree, the pattern and size of fragmentation on day 2, the size of the blastomeres on day 2 and day 3, the degree and pattern of fragmentation on day 3 (Table 2). Moderate intra-observer agreement was observed for the evaluation of the size of the pronuclei and the presence of a cytoplasmic halo on day 1, multinucleation on day 2, and the size of the fragments on day 3 (Table 2).

**Table 2:** Median (range) of kappa coefficient as measurement of intra-observer agreement in embryo evaluation

**Parameters**	**Median kappa coefficient (range)**
**Parameters day 1**	
Position of the pronuclei	0.60 (0.48-0.64)
Size of the pronuclei	0.54 (0.29-1)
Cytoplasmic halo	0.57 (0.33-0.77)
**Parameters day 2**	
Number of blastomeres	0.88 (0.82-0.94)
Degree of fragmentation	0.64 (0.59-0.68)
Size of blastomeres	0.71 (0.69-0.74)
Pattern of fragmentation	0.68 (0.46-0.86)
Size of fragments	0.60 (0.36-0.65)
Multinucleation	0.51 (0.32-0.79)
Orientation of the cleavage axes	0.85 (0.71-0.93)
**Parameters day 3**	
Number of blastomeres	0.87 (0.73-0.97)
Degree of fragmentation	0.67 (0.63-0.84)
Size of blastomeres	0.63 (0.45-0.70)
Pattern of fragmentation	0.61 (0.53-0.70)
Size of fragments	0.57 (0.46-0.70)

### Inter-observer variability

The inter-observer agreement was excellent (Table 3) for the scoring of the number of blastomeres on day 2 and day 3 and good (Table 3) for the evaluation of the orientation of the cleavage axes. The inter-observer agreement was moderate for the scoring of all the other embryo quality parameters except for the scoring of the size of pronuclei and the presence of a cytoplasmic halo where the inter-observer agreement was poor (Table 3).

**Table 3:** Inter-observer agreement indicated by the median (range) value of the kappa coefficient

**Parameters**	**Median kappa coefficient (range)**
**Parameters day 1**	
Position of the pronuclei	0.48 (0.14-0.73)
Size of the pronuclei	0.29 (0.07-0.66)
Cytoplasmic halo	0.31 (0.08-0.66)
**Parameters day 2**	
Number of blastomeres	0.88 (0.77-0.94)
Degree of fragmentation	0.52 (0.37-0.69)
Size of blastomeres	0.54 (0.34-0.71)
Pattern of fragmentation	0.49 (0.34-0.76)
Size of fragments	0.52 (0.30-0.83)
Multinucleation	0.51 (0.13-0.71)
Orientation of the cleavage axes	0.79 (0.62-0.93)
**Parameters day 3**	
Number of blastomeres	0.84 (0.70-0.94)
Degree of fragmentation	0.56 (0.21-0.74)
Size of blastomeres	0.50 (0.29-0.63)
Pattern of fragmentation	0.48 (0.33-0.69)
Size of fragments	0.51 (0.36-0.69)

### Training session: scoring of "trainees" versus "experts"

#### Inter-observer variability before training sessions (Table 4)

**Table 4:** Inter-observer variability among and between experienced embryologists and embryology trainees before embryology training sessions indicated by the median (range) value of the kappa coefficient

	**Experts vs trainees**	**Experts vs Experts**	**Trainees vs Trainees**
**Parameters day 1**			
Position of the pronuclei	0.27 (0.16-0.52)	0.28 (0-0.49)	0.29 (0.21-0.61)
Size of the pronuclei	0.31 (0.12-0.63)	0.27 (0.23-0.39)	0.14 (0.08-0.43)
Cytoplasmic halo	0.16 (0.03-0.67)	0.44 (0.36-0.52)	0.09 (0.02-0.10)
**Parameters day 2**			
Number of blastomeres	0.76 (0.70-0.88)	0.88 (0.82-0.88)	0.67 (0.63-0.79)
Degree of fragmentation	0.43 (0-0.70)	0.60 (0.53-0.61)	0.34 (0.28-0.52)
Size of blastomeres	0.25 (0.02-0.66)	0.52 (0.45-0.53)	0.43 (0.22-0.44)
Pattern of fragmentation	0.36 (0.19-0.42)	0.47 (0.45-0.48)	0.32 (0.20-0.49)
Size of fragments	0.22 (0.01-0.34)	0.52 (0.38-0.83)	0.04 (0-0.14)
Multinucleation	0.16 (0.07-0.32)	0.32 (0.27-0.64)	0.15 (0.07-0.16)
Orientation of the cleavage axes	0.60 (0.32-0.82)	0.79 (0.71-0.79)	0.38 (0.33-0.62)
**Parameters day 3**			
Number of blastomeres	0.52 (0.31-0.65)	0.86 (0.73-0.86)	0.44 (0.43-0.51)
Degree of fragmentation	0.18 (0-0.56)	0.35 (0.22-0.56)	0.05 (0.03-0.44)
Size of blastomeres	0.31 (0.06-0.70)	0.40 (0.39-0.50)	0.27 (0.23-0.48)
Pattern of fragmentation	0.28 (0.19-0.49)	0.47 (0.39-0.51)	0.23 (0.22-0.28)
Size of fragments	0.24 (0.07-0.57)	0.43 (0.41-0.45)	0.18 (0.02-0.26)

Inter-observer agreement was excellent when experts scored the number of blastomeres on day 2 and on day 3, but was only good when trainees scored the number of blastomeres on day 2. When the experts were compared with the trainees, there was a good agreement between groups with respect to the assessment of the number of blastomeres and the orientation of the cleavage axes on day 2. For the other parameters the inter-observer agreement was moderate, poor or very poor.

#### Inter-observer variability after training sessions (Table 5)

**Table 5:** Inter-observer agreement among and between experienced embryologists and embryology trainees after embryology training sessions indicated by the median (range) value of the kappa coefficient

	**Experts vs trainees**	**Experts vs Experts**	**Trainees vs Trainees**
**Parameters day 1**			
Position of the pronuclei	0.46 (0.24-0.56)	0.50 (0.45-0.56)	0.51 (0.40-0.59)
Size of the pronuclei	0.46 (0.19-0.77)	0.36 (0.29-0.60)	0.38 (0.24-0.68)
Cytoplasmic halo	0.53 (0.40-0.78)	0.61 (0.44-0.63)	0.69 (0.63-0.73)
**Parameters day 2**			
Number of blastomeres	0.85 (0.66-0.91)	0.77 (0.77-0.88)	0.79 (0.79-0.85)
Degree of fragmentation	0.45 (0.35-0.62)	0.68 (0.60-0.76)	0.55 (0.42-0.63)
Size of blastomeres	0.38 (0.10-0.54)	0.59 (0.59-0.64)	0.34 (0.27-0.51)
Pattern of fragmentation	0.41 (0.22-0.47)	0.48 (0.42-0.54)	0.43 (0.42-0.51)
Size of fragments	0.39 (0.21-0.59)	0.53 (0.35-0.54)	0.51 (0.43-0.52)
Multinucleation	0.39 (0.16-0.60)	0.53 (0.52-0.57)	0.25 (0.14-0.66)
Orientation of the cleavage axes	0.74 (0.58-0.89)	0.72 (0.71-0.86)	0.82 (0.64-0.82)
**Parameters day 3**			
Number of blastomeres	0.54 (0.48-0.65)	0.73 (0.73-0.84)	0.50 (0.48-0.56)
Degree of fragmentation	0.21 (0.10-0.47)	0.66 (0.53-0.78)	0.37 (0.29-0.65)
Size of blastomeres	0.45 (0.20-0.56)	0.52 (0.46-0.64)	0.32 (0.24-0.50)
Pattern of fragmentation	0.30 (0.15-0.47)	0.63 (0.53-0.66)	0.37 (0.35-0.49)
Size of fragments	0.42 (0.23-0.61)	0.54 (0.42-0.75)	0.53 (0.35-0.55)

Training sessions had a positive impact on inter-observer agreement, as becomes evident when comparing the data from table 5 (after training) with those from table 4 (before training). After training good instead of moderate inter-observer agreement was reached by experts for three extra parameters (cytoplasmic halo, degree and pattern of fragmentation on day 3) and by the trainees for two extra parameters (cytoplasmic halo and orientation of the cleavage axes).

## Discussion

To the best of our knowledge, this is the first study where intra- and inter-observer variability in embryo scoring has been assessed using multilevel images. The use of these multilevel images allows embryologists to assess embryo quality in the same way as an exploration at the inverted microscope, but without a limitation in evaluation time. Multilevel images allow the embryologist to evaluate among different focus planes within one embryo. Previous studies used 2D pictures which limits the evaluation to one focus plane in the embryo [8,9].

Intra-observer agreement was good to excellent for most parameters demonstrating that one individual embryologist is consistent in scoring the same embryo at different time points which is in line with previous publications [8,9]. Indeed, good intra-observer agreement has been reported with respect to the pattern of embryo fragmentation on day 2 (median kappa: 0.75), and with respect to number and size of blastomeres and degree of fragmentation on both day 2 (median kappa values of 0.79, 0.69 and 0.64 respectively) and day 3 (median kappa values of 0.66, 0.63 and 0.68 respectively) [9]. Other researchers [8] also demonstrated a good intra-observer agreement with respect to embryo scoring. However, their study was limited by the fact that only supernumerary embryos were used (not representative for daily embryology practice) and that different scoring systems with combined embryo quality parameters were used, whereas in our study embryos from routine practice were evaluated and all embryo quality parameters were scored individually.

Only moderate inter-observer agreement (median kappa 0.40-0.60) was achieved in our study for most embryo quality parameters on day 1, 2 and 3 that were not related to the number of blastomeres or the orientation of the cleavage axes. These results cannot be compared to poor inter-observer agreement (median kappa 0.24) in the assessment of embryo quality reported by some other investigators [8] for the reasons mentioned in the paragraph above. Moreover, the scoring of the most experienced person was considered to be the golden standard in that study [8]. However this consideration is not scientifically justified, as our study shows that experience is not necessarily linked to good inter-observer agreement. Our data demonstrating excellent inter-observer agreement for the number of blastomeres (day 2) and moderate inter-observer agreement in scoring the degree and pattern of fragmentation (day 2 and day 3) are in line with those from other investigators using a similar study design with individual assessment of embryo quality parameters [9]. However, evaluation of blastomere size (day 2), multinucleation (day 2) and blastomere number (day 3) had a lower inter-observer agreement in our study based on multilevel images than in the other study [9] based on 2D images. Embryo evaluation in 2D is limited by the fact that superimposed fragments may be invisible and are more difficult to assess. The inter-observer variability for some embryo characteristics, like the size of fragments or blastomeres, is possibly related to volume estimation which remains rather subjective. Furthermore the use of categorical variables, instead of continuous variables (percentages), may also contribute to the inter-observer variability.

Clearly, more consistency is needed in the evaluation of embryo morphology in order to investigate the impact of these parameters on embryo implantation. Both training and multilevel imaging may be beneficial to achieve that goal.

Inter-observer agreement was better among experienced embryologists than among trainees in our study and improved after training, not only among trainees but also among experienced embryologists. However, after training the kappa coefficient increased not for every parameter and inter-observer variability was still high among the trainees, suggesting that training sessions were not sufficient for the trainees in order to achieve reliable scoring of all embryo parameters. The need for training sessions in order to improve inter-observer agreement in the assessment of bovine or human embryo quality has been emphasized by other investigators [9,12]. Another idea to increase the inter-observer agreement [8] is to limit embryo scoring to be done by only one person, but this is not an option in the daily routine of larger centres.

The fact that our study was carried out in a single centre may be perceived as a limitation. Indeed, a multi-centre study is the ideal design to evaluate intra- and inter-observer variability in embryo assessment. However, multicentre design was not possible when our study was initiated because the technology used is still in development and only a few centres over the world work with the specific computer system and multilevel images used in our study. Furthermore, before a multicentre study is possible, the technical limitations arising when multilevel images are used have to be solved, to ensure that each centre uses this technology in the same way.

Our study design evaluated the intra- and inter-observer variability of individual morphological characteristics in embryo selection for intrauterine transfer. The final decision regarding the clinical use of the embryo was not considered since this is based on a combination of different characteristics.

In a next multicentre study, the variability in the clinical decision made for each embryo will be analysed in order to know if the variability in embryo scoring would influence this decision.

## Conclusion

In conclusion, the results of this study show that assessment of morphological characteristics of early stage embryos using multilevel images was marked by high intra-observer agreement and moderate inter-observer variability. Furthermore it demonstrates that training sessions were useful to increase inter-observer agreement.

## Competing interests

The authors declare that they have no competing interests.

## Authors' contributions

GP carried out the measurements of the study, performed the statistical analysis and drafted the manuscript. JD, SD and TD participated in the design of the study. CS participated in the design of the study and coordination and helped to draft the manuscript. All authors read and approved the final manuscript.
